# When safety for you means danger for me: the racial politics of carceral public safety discourse

**DOI:** 10.3389/fpsyg.2024.1347630

**Published:** 2024-07-04

**Authors:** Verónica Caridad Rabelo, Oscar Jerome Stewart, William C. Snowden, Sarah Fathallah

**Affiliations:** ^1^Management Department, Lam Family College of Business, San Francisco State University, San Francisco, CA, United States; ^2^School of Management, University of San Francisco, San Francisco, CA, United States; ^3^College of Law, Loyola University New Orleans College of Law, New Orleans, LA, United States; ^4^Independent Researcher, Oakland, CA, United States

**Keywords:** sense of safety, public safety, community safety, carceral logics, healing justice, abolition

## Abstract

Safety is a human right and universal need, and yet we as researchers and practitioners often take for granted the conditions that help people feel safe. In this conceptual review, we focus on factors that contribute to people’s sense of safety in service of understanding how, when, and where people feel safe. Moreover, we consider how race, power, and privilege shape people’s sense of safety and danger. In doing so, we highlight how public safety is not an objective or static reality but rather a political project that reflects dominant ideologies and serves state interests. We begin this conceptual review with a discussion of how public safety is a social construct whose meaning varies across time, space, and place. Next, we discuss three dominant ideologies that are embedded within collective public safety discourse: permanent bad guy syndrome, the victimization-fear paradox, and the politics of ideal victimhood. Together, these ideologies help to shape carceral public safety frameworks, which is the dominant paradigm in our culture. We then illuminate some of the underlying assumptions within carceral public safety frameworks and their implications for responses to public safety concerns, including elevating the safety concerns of dominant groups while criminalizing undesirable bodies, undermining stigmatized communities’ ability to access public safety and justice, legitimizing suspicion and surveillance, incentivizing carceral responses while diverting resources from safety promotion programs, and altering public spaces. In doing so, we highlight how carceral public safety frameworks reflect and reinforce existing injustices while also contributing to the stigmatization, marginalization, and manufactured precarity of social groups deemed undesirable and therefore unworthy of protection. We conclude with a discussion of alternative models of public safety which are rooted in life-affirming frameworks, which focus on improving people’s material conditions as a means of lessening and preventing the likelihood and impact of interpersonal violence.

## 1 Introduction

As we sat down to work on this article about how to cultivate public safety, our local mayor delivered a State of the City that revealed how “Oakland will aggressively pursue a comprehensive community safety strategy” (City of Oakland, 2023). Key features of the strategy include budgeting for six new police academies, expanding police foot patrols, and installing automated license plate readers (ALPRs). Thus, the heart of this strategy for enhancing public safety entails increasing police funding and presence throughout Oakland, California (City of Oakland, 2023). By responding to public safety concerns with calls for increased policing, Oakland’s incoming comprehensive community safety strategy is scaffolded by a carceral public safety framework. Carceral public safety frameworks are characterized by a reactionary orientation toward the punitive logics of the criminal legal system and the expansion of policing, such as by installing surveillance technologies, adopting militarized policing equipment, building new police academies, and expanding jails and prisons (e.g., Love et al., 2022). Carceral public safety frameworks assume that expanding policing will yield greater safety and less crime, despite the fact that law enforcement personnel are positioned to react to crime rather than prevent it. Moreover, surveillance technologies like ALPRs not only fail to deter crime, but also introduce new harms, given wrongful incrimination due to false positives (Díaz and Levinson-Waldman, 2020) and racial profiling given the disproportionate installation of ALPRs in low-income, Black, and Latinx neighborhoods (Maass and Gillula, 2015). Carceral public safety frameworks therefore favor punitive forms of social control (e.g., surveillance, institutionalization) to engineer a sense of public safety for some (i.e., white ruling class) at the expense of actual safety for everyone else.

Carceral public safety frameworks rely on the state apparatus to define crime, identify threats, and allocate resources in service of eliminating these threats, generally reactionarily but also preemptively. Carceral public safety frameworks date at least as far back as former President Johnson’s 1965 declaration of the War on Crime, which paved the way for the subsequent Presidential administration’s War on Drugs (Hinton, 2015). Throughout the decades since then, carceral approaches have not systematically addressed the material conditions that allow for public safety. Rather, they have primarily been concerned with achieving a *sense* of safety for a select few—namely, the interests and property of the dominant class of capitalists (Robinson and Scaglion, 1987; Uchida, 1993) and those interested in upholding whiteness (Burton, 2015; Kaba and Ritchie, 2022). *Sense of safety* is a subjective feeling of security someone has in a defined space, such as a community, neighborhood, or city, at a given point in time (Collins and Guidry, 2018). In a society marred by carceral public safety ideology, subjective feelings of safety among privileged groups are often fueled by discomfort and fear of dissimilar people and unfamiliar situations (Lupton, 1999) as well as by racially biased news coverage (Jackson, 2019; Baranauskas, 2020). Actual public safety, on the other hand, is concerned with creating conditions so that violence and crime are less likely to occur, primarily through ensuring that people are able to meet their basic needs. Actual public safety involves not only being free from “physical, material or moral threats” (Maurice et al., 1997, p. 181), but also meeting the community’s needs to thrive. Kaba and Ritchie (2022) explain that safety is not a static, stable state, nor can it be understood in universally shared terms. In actuality, safety depends on social relations and is contextual, given how our respective safety is contingent on “our relationship to others and our access to the resources we need to survive” (p. 276). In depriving people of the resources and relationships necessary to survive and thrive, carceral approaches *undermine* public safety.

Carceral approaches cannot sustain long-term safety for all members of our society, especially those who are criminalized (i.e., most vulnerable to state surveillance and policing). It’s possible that carceral approaches enhance a *sense* of safety for the powerful and privileged few, without materially improving *actual* safety for all. Carceral approaches not only fail to promote safety, they also exacerbate harm for criminalized communities and their loved ones, often for subsequent generations to come, given the spillover and crossover consequences of carcerality (Love et al., 2022). Carceral public safety frameworks divert resources away from programs that have been proven to decrease harm and enhance public safety, such as affordable and equitable access to housing, harm reduction services, healthcare, food security, and high-quality education (e.g., Hamaji et al., 2021; Page and Woodland, 2023). Carceral frameworks also prevent people from forming, maintaining, and repairing the social connections needed to build and nurture healthy communities. This divestment from social ties and safety nets in service of investment in carceral structures is a trend that we are witnessing not just in Oakland but also across the United States (Norris, 2020; Hamaji et al., 2021). This worrisome state of affairs begs some questions:

1. Which communities are prioritized in carceral public safety interventions? Which communities are invoked as public safety threats?
2. How do carceral public safety frameworks reflect, reinforce, and amplify existing injustices?
3. How do carceral public safety interventions impact communities who are most vulnerable to surveillance and policing?
4. What are some alternative interventions that would promote public safety for everyone, not just those with the greatest privilege and access to resources?

To address these questions, we first consider how public safety is socially constructed, highlighting three ideologies that shape carceral public safety discourses: permanent bad guy syndrome, the victimization-fear paradox, and the politics of ideal victimhood. We also illuminate some of the underlying assumptions within carceral public safety discourse and their implications for responses to public safety concerns, including elevating the safety concerns of dominant groups while criminalizing undesirable bodies, undermining stigmatized communities’ ability to access public safety and justice, legitimizing suspicion and surveillance, incentivizing carceral responses while diverting resources from safety promotion programs, and altering public spaces. We conclude with a discussion of alternative models of public safety which are rooted in an ethics of care and justice.

## 2 The social construction of public safety

Social constructionism argues that knowledge is a collection of ideas that are shaped by social interactions and power relations (Berger and Luckmann, 1966; Burr, 2015). Knowledge is not an objective, external truth or reality. Rather, it is shaped by dominant discourse. Crime is one such example of a social construct shaped by dominant discourse, insofar as the definition of crime changes across time and location, dominant groups control which actions are criminalized and how they are persecuted, different groups are not held to the same standards for “committing” crimes (i.e., “crime” propensity is not actually higher among racialized minorities, but criminalization is), and people’s perceptions of crime often differ from reported crime statistics (e.g., Quillian and Pager, 2001). Legal frameworks generally define crime as “an act or omission constituting an offence (usually a grave one) against an individual or the state and punishable by law” (Oxford English Dictionary, 2024). This definition of crime aligns with orthodox criminology, which unquestioningly accepts legal/judicial approaches to understanding crime (Michalowski, 2016). Such an understanding of crime ignores, for example, why many societal dangers that compromise people’s actual safety and security (e.g., dangerous working conditions, wage theft, and financial schemes targeting lower income racial minorities) are either not labeled as crimes or not enforced as such (Robinson, 2000).

For example, in June 2021, a video circulated of a man stealing less than $1,000 worth of goods from Walgreens. The incident was mentioned multiple times across all major news outlets as proof of San Francisco’s lack of public safety (Legum, 2021). The man was subsequently convicted of felony grand theft, sentenced to 16 months in prison, and 1 year of probation (Cassidy, 2022). Not a year before that, Walgreens resolved a class action lawsuit stemming from 7 years of wage theft from its employees for $4.5 million, which shockingly only represents “approximately 22% of the potential damages” (Shubb, 2020). This devastating crime roused only one story in a major news outlet (Legum, 2021). A singular instance of petty theft for less than $1,000 generated more news coverage and criminal punishment than a seven-year long wage theft scheme totaling more than $4.5 million, for which nobody was actually held accountable. As Jackie Wang (2018) notes, “socioeconomic conditions are what cause crime as well as what determine which kinds of activities get counted as criminal” (p. 63). Perceptions of crime, threat, and safety thus vary across social groups and therefore are not rooted in a shared universal reality.

Just as crime is socially constructed, so too is public safety. The United States government spends more than $100 billion per year on what it categorizes as public safety, the majority of which is devoted to maintaining and expanding policing (Friedman, 2022). The mutual constitution of public safety and carceral logics is also reflected in the visual landscape surrounding public safety. For example, the landing page for the San Francisco State University Division of Campus Safety features photographs of uniformed law enforcement officers and a letter from the Chief of Police, thus communicating the inextricable tie between public safety and policing. These images are neither accidental nor idiosyncratic, but rather reflect a larger pattern of painting police as the face and bastion of public safety (Friedman, 2022). Such imagery reflects the faulty assumption that law enforcement officers possess the willingness and capability to prevent harm, and that they themselves do not compromise public safety (Owens and Ba, 2021). This faulty logic is reinforced through common phrases that are typically associated with policing bodies, such as “protect and serve,” despite both the Supreme Court ruling that the police do not actually have a constitutional duty to protect constituents from harm (Greenhouse, 2005) and the origins of police protection being firmly rooted in the protection of capital, resources, and people who were enslaved and treated as property (Robinson and Scaglion, 1987). The enduring nature of these visual and linguistic associations between policing and protection—despite the fact that the police do not have an occupational or legal responsibility to protect the public, nor a history of doing so—reflects *public safety hegemonic discourses*.

Hegemonic discourses are the norms, values, and ideologies of dominant groups which masquerade as “common sense” and therefore offer the illusion of consent (Stoddart, 2007). Hegemonic discourses function insidiously to guide people into internalizing, adopting, and enforcing practices that preserve and strengthen the status quo (Stoddart, 2007). We draw from a hegemonic discursive perspective to contest the taken-for-granted nature of public safety and instead illustrate how public safety is a political project that mirrors and serves dominant ideologies. In doing so, we illuminate how popular understandings of public safety are rooted in a mythos which ultimately serves to protect the status quo and prioritize state interests above community well-being.

## 3 Hegemonic public safety discourse

We explore three factors that shape public safety discourse, people’s sense of safety, and the interplay between the two: (1) permanent bad guy syndrome, (2) victimization-fear paradox, and (3) politics of ideal victimhood.

### 3.1 Permanent bad guy syndrome

First is the notion of *permanent bad guys* (Norris, 2020, p. 91), whereby some people (or groups) are regarded as inherently “unsafe,” “bad,” or “evil.” This adherence to the notion of permanent bad guys contributes to essentialist beliefs about the origins of “dangerous” behavior (i.e., naturally dangerous people), and also draws attention to individual behavior (vs. social systems) as the root cause of danger and harm in our communities (Norris, 2020). Below we highlight five of the most pervasively stereotyped permanent bad guys.

First, the myth of the “superpredator” constructs the archetype of the young, violent, remorseless, animalistic Black teenager. Originally mentioned in a then-obscure criminology paper, superpredator as a term and mythology became viral across mass media in the 1990s, from news stories to police reports to political discourse (Jennings, 2014), including a 1996 speech by Hillary Clinton (C-SPAN, 2016). The racialized crime mythology driving fear of Black teenagers matures into fear of Black men. Just as the myth of the superpredator paints young Black men as inherently dangerous, so too does racialized crime mythology shape Black men as the prototypical permanent bad guy more generally. Research has found that white people’s perceptions of neighborhood crime are positively correlated with the percentage of young Black men residing in that neighborhood, even while controlling for reported crime rates (Quillian and Pager, 2001; Pickett et al., 2012). These racist misconceptions both reflect and are reinforced by dominant imagery in mass media and popular culture that equate Blackness with criminality (e.g., Robinson, 2000; Quillian and Pager, 2001). This imagery is deeply embedded in U.S. culture and psyche; the country needed to dehumanize and criminalize Blackness to justify slavery, slavery’s extension via the convict leasing system, the Black Codes, Jim Crow, and then mass incarceration (Alexander, 2010; Burris-Kitchen and Burris, 2011; Snowden, 2022). Since the creation of chattel slavery in the United States, constructions of crime have relied on pejorative mythologies of Blackness, and in particular Black men. Thus, racial stereotypes drive (mis)perceptions of, and desired responses to, crime. In this way, crime is racialized, Blackness is criminalized, and the specter of the permanent bad guy gives rise to policies that serve to eliminate him from public view and existence.

A second archetype of the permanent bad guy stems from xenophobia, especially against Black or brown-skinned people who are presumed to be non-American. As an example, people who are Arab, Muslim, and/or perceived as such are subject to permanent bad guy syndrome as a result of being interpolated through the gaze of “war on terror discourse” (Kurwa, 2019, p. 115). Black people, Muslim people, and especially Black Muslim men are selectively subject to hyperpolicing and organized surveillance (Mauleón, 2018) due to their consideration as inherent threats to public safety and national security.

A third group subject to permanent bad guy syndrome under carceral public safety discourse is trans people. Many trans people (especially transgender women) are portrayed as sexual predators, and their supposedly predatory nature is used to justify laws that exclude trans people from public life in the name of protecting the safety of women and children (Bagagli et al., 2021). Ironically, even though transgender people (especially transgender women of color) are rendered as inherently dangerous in the social imagination, they themselves suffer disproportionate rates of violence, including intimate partner violence and police brutality (Carpenter and Marshall, 2017). Similarly, young trans people are far more likely than cisgender teenagers to experience violence in school restrooms (Murchison et al., 2019), in stark contrast to the myth of the transgender “bathroom predator” (Schilt and Westbrook, 2015). Characterizing trans people as inherently predatory and dangerous both reflects and stokes cisgender people’s fears of trans people. These unwarranted fears enable policies that result in the surveillance, hypercriminalization, and isolation of trans people, and in the process overlook or even thwart trans people’s access to public safety.

Fourth, many people struggling with mental illness are also rendered permanent bad guys. Society often represents them as inherently dangerous despite the fact that they are far more likely to endure, rather than commit, violence (Pescosolido et al., 2019). This is reflected in the tendency for 911 gatekeepers to dispatch paramedics to “medical” emergencies and law enforcement officers to “behavioral” emergencies (Townsend et al., 2023). However, law enforcement officers receive little mental health first aid training (if any) and demonstrate poor performance when it comes to identifying mental illness and supporting people in crisis (Townsend et al., 2023). Nearly one in four people who are murdered by police have prior experience with mental illness, making this group seven times more likely to be murdered by police compared to people without mental illness diagnoses (Saleh et al., 2018; Townsend et al., 2023). These disparities are even starker when accounting for race/ethnicity (Saleh et al., 2018).

A final archetype of permanent bad guys is people experiencing poverty, especially homelessness. Law enforcement, news outlets, and social media all typically scapegoat people enduring housing insecurity as criminal and unlawful (e.g., Barak and Bohm, 1989; Robillard and Howells, 2023). Homelessness is indeed characterized by violence, albeit in the reverse direction; the structural conditions of poverty that allow homelessness to exist and persist are symbolically and materially violent (Fischer et al., 2008; Roy et al., 2014), not to mention the added risk among those who are also trans, mentally ill, and/or members of the Global Ethnic Majority (i.e., people who identify as “Black, Asian, Brown, dual-heritage, indigenous to the global south, and or have been racialized as ethnic minorities”; Campbell-Stephens, 2020, p. 1). Moreover, people who are unhoused are at a significantly increased risk of being victims of violent crime (Fischer et al., 2008; Nilsson et al., 2020; Kushel et al., 2023). This risk of victimization is likely underestimated, as state-sanctioned violence during eviction processes—wherein people are stripped of their most basic form of safety and dignity (Desmond, 2016; Barocas et al., 2023)—is not considered as either a form of violence nor a threat to public safety. And yet, dominant discourse and public policy routinely criminalize homelessness in the name of public safety, despite a lack of evidence that unhoused people threaten public safety (Rankin, 2019). Consequently, because society views people who are unhoused as permanent bad guys, crimes *against* them are not widely considered in discourses of public safety. Indeed, over the past decade, nearly one-third of victims assaulted by the San Francisco Police Department have been homeless, despite them comprising less than 3 % of the general population (Baustin and Barba, 2023).

Taken together, the archetypes of the permanent bad guy shapes people’s sense of public safety as well as their desires for how public policies ought to account for the people and communities who are deemed threats. Permanent bad guy syndrome is widespread in the general public as well as among powerful state actors such as policymakers and law enforcement officers.

### 3.2 Victimization-fear paradox

Research shows that declining crime rates do not necessarily increase people’s sense of public safety (Rader, 2017; Norris, 2020). Thus, a third factor driving people’s sense of safety can be accounted for by the *victimization-fear paradox,* whereby people’s *fear* of being victimized is not strongly, consistently, or linearly correlated with their *actual likelihood* of experiencing harm (LaGrange et al., 1992; Zacharia and Yablon, 2022). There are a few reasons for this disconnect. Observing or hearing about crime is more common than experiencing it oneself (Zacharia and Yablon, 2022). Information sources can therefore shape people’s risk assessment. Specifically, news outlets report on crime in ways that shape how people understand levels of and their proximity to crime (Sacco, 1995; Romer et al., 2003). It is possible that these fears can be further stoked by right-wing populist governance (Villar and Magnawa, 2022; Wang and Catalano, 2022), conservative political talking points (Gramlich, 2020), and racial bias in the media coverage of crime (Jackson, 2019; Baranauskas, 2020).

In this way, one’s fear (and, by extension, sense of safety) may be racialized; for instance, members of dominant groups—such as white people and those with greater proximity to whiteness—may confuse fear and anxiety for danger (Jackson, 2019), and therefore have a distorted, lower threshold for detecting threats (Wang, 2018). This racialization of threat (French and Monahan, 2020) results in responses which dehumanize, surveil, isolate, and even eliminate people of color and people who are unhoused, whose mere existence activates white discomfort (Rankin, 2016). These responses range from interpersonal behaviors to public policy interventions designed to make certain people (e.g., white people) feel safer. We can see evidence of racialized threats manifest in real-time on social media platforms that enable and encourage people to issue, receive, and discuss alerts related to local crime, including Nextdoor, Citizen (formerly called Vigilante), and Amazon Ring’s Neighbors (Molla, 2019). These apps allow and amplify racial profiling and vigilantism under the guise of building “public safety networks.” However, these networks amount to echo chambers, given how these apps are particularly popular among white, affluent, property-owning residents, many of whom are newcomers to gentrifying neighborhoods (e.g., Lowe et al., 2022). In sum, social media can become a hotbed for the victimization-fear paradox, given how certain apps and features rely on—and even amplify—affluent white people’s fear of experiencing crime, in spite of evidence demonstrating decreasing crime rates (Molla, 2019).

### 3.3 Politics of ideal victimhood

A third factor contributing to one’s sense of public safety involves the *politics of ideal victimhood*, which determine who is deserving of protection, and from whom. Ideal victims must be law-abiding with no criminal history, strangers to their perpetrators, and demonstrably nonthreatening, whether very old or very young, weak, and/or disabled (Christie, 1986; Long, 2018). These criteria demarcate ideal victims and render them as deserving of help and safety. Because anti-Black racism plays such a central role in the public’s social constructions of a sense of safety, crime, and f policing, an important defining feature of ideal victimhood is white femininity. Public safety in the U.S. has always been designed to protect white women from the perceived menacing threat of Black men (Feimster, 2009; Smångs, 2017; Armstrong, 2021). The constant reinforcement of Blackness as an affront to white femininity (De Welde, 2003) centers not only whiteness but also cisgender womanhood in discourse concerning who is deserving of safety. Taken together, public safety is a social construct, with hegemonic public safety discourses privileging the dominant capital-owning classes, whiteness, and women—which ultimately strengthens the intersecting structural violence “of capitalism, white supremacy, and heteropatriarchy” (Phipps, 2021, p. 86).

These criteria for ideal victimhood also limit the ability of “unideal” victims to seek support, effectively rendering them “nonvictims.” This is the case for people who are incarcerated, the vast majority of whom are survivors of violence themselves (whether prior to and/or during their imprisonment) yet not deemed deserving of protection or safety due to their detention (Spade, 2014). It also includes people who are unhoused, as overt signals of poverty transform victims into “bad guys” quickly and often. The politics of deservingness rely on the ideal victim/ideal enemy binary, whereby ideal enemies are social outcasts who are unlikable, easy to scapegoat, and thereby deserving of isolation and retribution (Christie, 1986). The twin archetypes of ideal victims and ideal enemies serve to reinforce other essentialist binaries, such as good vs. evil, human vs. animal, victim vs. perpetrator, and safety vs. danger. The criteria that constitute ideal victimhood appeal to dominant notions of innocence and essentially establish the ideal victim as “nonthreatening to white civil society” (Wang, 2018, p. 265). Put differently, victimhood is less about “being” a victim—i.e., experiencing victimization—and more about “becoming” a victim—i.e., successfully performing victimhood and being conferred victim status through the white gaze (Long, 2021; Goodmark, 2023). The politics of ideal victimhood determine who needs protection and, by extension, whose safety will be considered, prioritized, and deemed worthy of investment.

### 3.4 Summary

Public safety is not a static state, nor is it rooted in a universally shared understanding of safety and crime. Rather, various ideologies shape people’s sense of public safety, including permanent bad guy syndrome, racialized crime mythology, the victimization-fear paradox, and the politics of ideal victimhood. These ideologies are also influenced by people’s social identities (e.g., gender, sexuality, race, ethnicity, etc.) and socialization (e.g., social media vigilantism, mass media bias). Critically examining the factors that shape one’s sense of safety, as well as feelings around who is deemed deserving of safety, reveals and reifies the hegemonic discourses surrounding public safety, which are ultimately carceral in nature. In the next section, we detail some of the consequences of carceral public safety discourse and illustrate how these frameworks serve to maintain and legitimize inequitable power relations.

## 4 The consequences of hegemonic public safety discourse

Public safety discourse is insidious, given how it appeals to the universal need for security and assumes consensus in terms of how to promote and preserve public safety. Upon closer examination, cultivating a sense of public safety is a political project that serves vested power interests and carries important implications for community relations. We now detail eight of these implications. First, we discuss how hegemonic public safety discourses shape community relations by creating the categories of safe/unsafe, harm-doer/harmed, and deserving/undeserving, then sorting people into these binaries. This category creation functions to (1) elevate the safety concerns of dominant groups while downgrading those of marginalized groups, (2) criminalize undesirable bodies, (3) undermine stigmatized communities’ ability to access public safety and justice, and (4) essentialize some groups as “dangerous” to legitimize suspicion and surveillance. We then discuss how hegemonic public safety discourses alter the public safety apparatus itself, as deployed by the state. These alterations are deployed to ensure that the above social categories are maintained, and are upheld by practices and policies that (5) displace focus from structures to individuals, (6) incentivize carceral responses, (7) divert resources from safety-promoting programs, and (8) alter public spaces.

### 4.1 Elevate the safety concerns of dominant groups while downgrading the safety concerns of marginalized groups

Hegemonic public safety discourses elevate the safety concerns of dominant groups—namely, those who are white, cisgender, heterosexual, able-bodied, documented, formally educated, and/or economically resourced. Members of these groups will find it easier to fulfill the criteria for ideal victimhood. Missing White Woman Syndrome typifies such privilege, as missing white women and girls receive disproportionate media attention compared to missing women and girls of the Global Ethnic Majority (Lucchesi, 2019). Members of dominant groups are also more likely to be surveyed and listened to when expressing public safety concerns, whether while filing police reports or commenting during municipal town halls. As a result, their voices overshadow those of people with less power and privilege who are also disproportionately vulnerable to harm and danger, such as trans women of color, people suffering homelessness, disabled, and/or Mad (an identity label reclaimed by people who have been assigned psychiatric diagnostic labels and/or have survived psychiatric incarceration). In this way, hegemonic public safety discourses communicate which people are deserving of safety, protection, and a sense of urgency. Hegemonic public safety discourses center the protection of not only privileged people, but also their property, at the expense of the safety of the criminalized and policed. For example, in California, carceral public safety rhetoric decries the (perceived) increase in robberies since the COVID-19 pandemic as evidence of declining public safety. It remains unclear, however, *who* is rendered unsafe by this alleged uptick in theft, since the overwhelming majority of state-categorized robberies target businesses, ranging from banks to convenience stores to retail organizations (Bonta, 2022). In response, Governor Newsom of California has deployed 120 California Highway Patrol officers to the City of Oakland, a decision that has the potential to enable greater racial profiling and criminalization of Black and brown residents while also investing state funding toward policing instead of projects that can promote actual safety for vulnerable groups.

### 4.2 Criminalize undesirable bodies

Public policies are one vehicle for helping powerful groups control the terms and conditions of belonging into social life. Hegemonic public safety discourses reflect this value system that dictates who deserves to exist in public space. The United States has an extensive history of vagrancy legislation designed to regulate whether and how people can exist in public, such as Jim Crow, anti-Okie, and “ugly” laws (Fisher et al., 2015). In California, for example, there are more than 500 laws that regulate people’s existence in public spaces, including restrictions on standing, sitting, resting, sleeping, asking for money, and sharing food (Fisher et al., 2015; Norris, 2020, p. 142). These behaviors are criminalized not because they pose a threat to bystanders’ safety, but because they threaten privileged people’s ability to feel comfortable and compromise their purchasing power. If anything, restrictions created by vagrancy legislation compromise the health and safety of the people who are surveilled, policed, and displaced under these policies. This is exemplified by the tendency for municipal governments to destroy the homes, property, and care networks of curbside communities in anticipation of tourists visiting for special events, such as when San Francisco hosted the Asia-Pacific Economic Cooperation (APEC) forum (Tong and Henderson, 2023) and as New Orleans prepared to host the Super Bowl (McNeil, 2023). When municipal leaders conduct “sweeps”—a euphemism to describe the involuntary displacement of people experiencing homelessness—people suffer consequences for years to follow. City-funded involuntary displacement is predictive of decreased safety, as well as sense of safety, in the form of increased hospitalizations, more frequent and severe drug overdoses, and premature death over the following decade (Barocas et al., 2023) as a result of losing access to one’s belongings, social networks, and kinship ties (Goldshear et al., 2023). The criminalization of poverty to appease white comfort therefore incurs violent, even lethal, consequences for those relegated to the margins of social life.

Similar policies and practices restrict the movement, freedom, and existence of other groups who are stigmatized or otherwise deemed unworthy in the public eye. For example, everyday surveillance and policing is such a common experience for trans women that they have deemed the phenomenon “walking while trans” (Carpenter and Marshall, 2017). Similarly, youth of the Global Ethnic Majority are treated as undesirable in public because they instill outsized fear in property-owning, primarily white residents. Consequently, police have hyperpoliced these youth, historically and through the present day (e.g., Vera Sanchez and Adams, 2011). Similar attempts to extricate Black youth from the public eye due to “safety concerns” are evidenced by city removal of public basketball courts (e.g., Haggerty, 2021; Saldanha, 2021).

In summary, hegemonic public safety discourses help determine which people deserve to exist in public, as well as the consequences for those who dare to defy these attempts at social regulation. The policies that dictate these consequences send a clear message about which types of people are permitted to belong to public life and which bodies ought to be removed (Norris, 2020). These policies enact systemic violence—symbolically, psychologically, physically, materially, and structurally—against vulnerable communities under the guise of public safety.

### 4.3 Undermine stigmatized communities’ ability to access safety and justice

Hegemonic public safety discourses make it difficult for some groups to access safety, particularly when they incur social stigma and/or are deemed non-ideal victims (or even non-victims). The politics of deservingness rely on the twin archetypes of ideal victim (white woman) and prototypical perpetrator (Black man). These archetypes function in tandem to render Black men as perpetual suspects (Long, 2018), even when they are trying to seek support after experiencing harm themselves (Long, 2021). In this way, hegemonic public safety discourses compromise marginalized people’s ability to seek help when they do experience harm.

Other stigmatized communities also encounter difficulties when trying to seek support and safety, such as people who are undocumented and sex workers, especially those who are queer, trans, and/or people of the Global Ethnic Majority (Singer et al., 2021). Their criminalized existence renders them undeserving of safety, care, and justice in the eyes of the law. Similar challenges abound even within organizations intended to support underserved communities. In the 1980s and 1990s, many feminist and queer health clinics were exclusionary toward clients who were transgender, sex workers, and/or active substance users (Page and Woodland, 2023, pp. 23–24), thus exacerbating precarity amongst some of the most vulnerable members of society in the name of safety for those deemed more “respectable” and worthy of support. Stigma fuels discrimination and deprives vulnerable community members from opportunities to access support and safety. Hegemonic public safety discourses reflect and reinforce such stigma, and compromise the wellbeing of people living and working in unsafe conditions.

### 4.4 Essentialize some groups as dangerous to legitimize suspicion and surveillance

Hegemonic public safety discourses reinforce the essentialism of some groups as being inherently dangerous. When social groups are portrayed as innately dangerous, it becomes easier to dehumanize them and justify the need to remove them from public life. As a result, hegemonic public safety discourses help to legitimize suspicion toward and surveillance of “dangerous” people. For instance, the social construction of Muslim people as innately suspicious and dangerous was made abundantly clear when one of Trump’s first acts as president was signing Executive order 13769, dubbed the “Muslim Ban” (Mauleón, 2018). This policy decision reflected the anti-Muslim hate and fearmongering that both preexisted among Trump’s supporters and then was further stoked and provoked amidst his presidential campaign.

Some other social identity groups that are essentialized as inherently dangerous and suspicious include people who are Black, immigrants, trans, mentally ill, unhoused, and/or those who are assumed to hold any of these identities, regardless of how they actually identify. Members of these groups are often rendered permanent bad guys and are surveilled, criminalized, policed, and prosecuted in the name of prioritizing the public safety of dominant groups (namely, white/cisgender people, especially women and children) and their property. This systematic suspicion and surveillance become heightened when dominant group members feel anxious or uncomfortable, and therefore “threatened.”

This racialization of threat can be found within many platforms designed to promote public safety. For example, in 2011, the University of Michigan Department of Public Safety issued a clime alert which described the suspect as “bald or with dread locks [*sic*]” and wearing a sweatshirt that was “orange, red, or black” (Green, 2011). This description, at once contradictory and vague to the point of futility, was excessively inclusive and legitimized the suspicion of any Black men fitting the descriptors. In circulating this alert to tens of thousands of people, so-called Public Safety leaders essentially cast a net so wide as to scapegoat, criminalize, and therefore endanger countless Black residents fitting any of the many descriptors within the crime alert. David Green (2011) described this event as a form of *viral racism* “because, like a virus, these descriptions spread through the University system and thus perpetuate a gendered formation of blackness that’s inherently criminal and deviant. This viral racism is a subtle practice of racial profiling that’s legally and federally protected in the name of campus ‘safety’ vis-à-vis the Clery Act.” This crime alert was not idiosyncratic, but rather part of a larger trend of overly broad suspect descriptions that can serve to justify the surveillance, racial profiling, and inherent dangerousness of Black and Latino people (Savin, 2018). The weaponization of discomfort in the face of manufactured racial threats can be deadly, such as when George Zimmerman, a volunteer “neighborhood watch” patrolman, murdered Trayvon Martin, a child, in his gated community. Social media platforms such as Citizen, Nextdoor, and Ring operate as virtual gated communities (Kurwa, 2019), whereby neighbors can encourage profiling and surveillance by sharing information about “suspicious” and “sketchy” people in their neighborhoods (Harshaw, 2015). These exchanges are often characterized by vague descriptions and dubious grounds for suspicion, thereby reducing Black neighbors to threats.

### 4.5 Displace focus from structures to individuals

Hegemonic public safety discourses locate the site of safety and danger at the intrapersonal or interpersonal level of analysis at the expense of acknowledging the culpability of institutions, such as violence inflicted from corporations (e.g., environmental pollution) and the state (e.g., police brutality). Characterizing individual people as “safe” or “dangerous” is not only essentializing, but also obfuscates how structural oppression and institutional violence harm members of marginalized groups (e.g., Wang, 2018; Long, 2021). This violence often comes from the very institution that the carceral public safety discourse has crafted as the purveyor of safety - the police. Misconduct is rampant among police. For example, police officers engage in sexual misconduct regularly, assaulting and harassing people during traffic stops, people who have witnessed or been victims of crime, people participating in sex work, minors, and even their own relatives (Stinson et al., 2015, 2020; Mennicke and Ropes, 2016).

This displaced focus from structures to individuals can lull dominant group members into a sense of safety at the expense of groups that have been extracted from the public eye using the violent means of structural oppression, such as mass incarceration. As described by Jackie Wang (2018), “The violent foundation of U.S. freedom and white safety often goes unnoticed by those who live in relative safety” (p. 287). Hegemonic discourses appeal to people’s common sense understanding of the world to obfuscate harm, such as how California refers to policing personnel as “peace officers” despite their increasingly militarized equipment and practices, as well as the documented ways that so-called peace officers have inflicted psychic and physical violence upon constituents (Rodríguez, 2012). Locating the site of public safety management within the individual not only distracts from the role of structural harm, but also empowers dominant group members to embody and enact policing tactics, which we discuss more below.

### 4.6 Incentivize carceral responses

Hegemonic public safety discourses invite, inspire, and incentivize carceral responses to public safety concerns. Research shows how the criminalization of Blackness is associated with the desire for expanding carcerality, such as greater spending on crime prevention and response as well as more punitive policies to control crime (Mancini et al., 2015). When scaled up, these desires for expanded carcerality turn crime control into an industry (Norris, 2020, p. 28) rather than an effective and equitable approach to safety promotion. Such desires and practices amount to what Norris (2020) calls the fear-based model, which “defines safety only in terms of being free from crime and criminals, which is limited, and limiting” (p. 9). Under the fear-based model, there are four practices that drive efforts to instill safety in communities: “systemic deprivation, extensive and expensive systematic suspicion, cruel punishment, and often-permanent isolation from the rest of society” (p. 10). Although these interventions are lauded and implemented to instill safety, fear-based approaches fail because police generally fail to prevent crime or promote safety. Rather, it is the vast resources and safety nets to which the privileged and powerful members of society have access that contribute to their actual safety, even if their sense of safety is correlated with police expansion.

Moreover, the fear-based model of public safety justifies and expands policing and prosecution for communities that are already surveilled, criminalized, and otherwise rendered vulnerable due to state violence and neglect. The fear-based model, upheld by hegemonic public safety discourses, construes Black people as “symbolic assailants” which then justifies their frequent subjugation to stop-and-frisk (unwanted and unwarranted searches/seizures), intimidation, displacement, and even assault (Brunson and Miller, 2006). These consequences were magnified amidst the proliferation of the “superpredator” myth, whose dehumanizing discourse quickly ushered in extreme sentencing practices, such as juveniles to life without parole, that disproportionately targeted young Black men and boys (Mills et al., 2016). The pipeline from dehumanizing discourse to dehumanizing carceral harm is further made apparent when examining disparities in capital sentencing outcomes. Archival research shows that jurors are more likely to advocate for the death penalty against Black defendants who have more stereotypically Afrocentric physical features (Eberhardt et al., 2006).

Policing practices in the name of public safety therefore constitute injustice for people who are criminalized, given the double standards that subject them to scrutiny and harm. Moreover, research shows that mass incarceration does not actually reduce or control crime (National Research Council of the National Academies of Science, 2014). That the expansion of policing may make some groups feel safer while exposing other groups to greater harm/danger illustrates how public safety programs prioritize the interests of “ideal” victims of crime (who are also “ideal” members of society). Moreover, these ideal victims serve as a foil to justify the removal of “undesirable” others from public life. As Jackie Wang (2018) notes, “Historically, appeals to the sexual safety of women have sanctioned the expansion of the police and prison regimes while conjuring the racist image of the black male rapist” (p. 271). Such “carceral creep” (Kim, 2020) permeates many public policy approaches to public safety concerns. For example, the 1994 Violence Against Women Act led to the investment of billions of dollars into policing and prosecution (Goodmark, 2023), part of a larger trend of carceral feminism (Bernstein, 2010) whereby carceral logics are used to justify the expansion of policing in the name of safety without meaningfully reducing or preventing actual rates of violence and harm.

### 4.7 Divert resources from safety-promoting programs

Investment in carceral responses occurs in tandem with divestment from social services that help promote actual public safety, namely by minimizing and preventing harm in our neighborhoods and institutions (Wang, 2018; Norris, 2020, p. 88; Hamaji et al., 2021). Historically, social services such as public education, welfare spending, and youth services have contributed to the long-term safety of communities. Yet, spending on these services, particularly for those most in need, has decreased as carcerally-aligned spending toward public safety has expanded (Moffitt, 2015; Beck and Goldstein, 2018). Expanding policing in the name of public safety is costly for communities. For instance, in 2023, New York City subway police officers were awarded $155 million in overtime pay—a nearly 40-fold increase compared to the $4 million awarded in 2022—despite just a two-percent reduction in “major crimes” during this time (Ostadan, 2023). Additional costs of community disinvestment include those incurred from the criminalization and subsequent mass incarceration of Black and Latinx communities (Western, 2006; Alexander, 2010).

### 4.8 Alter public spaces

Hegemonic public safety discourses also justify the transformation of public spaces in ways that can undermine actual public safety. Regarding a housing project in Chicago, law enforcement officers maintained that increased vegetation would stoke fears among residents, whereas in actuality the residents felt that greater vegetation and more intentional landscaping would enhance their sense of safety (Kuo et al., 1998). Thus, when conversations about public safety center on the desires of law enforcement, the safety concerns and needs of other members of the community are not prioritized or taken seriously, sometimes to the detriment of their sense of safety as well as actual safety.

At times, people’s taxpayer dollars fund the very infrastructure that makes public spaces less hospitable and more dangerous. Hostile architecture refers to environmental features “with the aim of discouraging specific uses of public space, frequently with the goal of pushing a particular population out of public space entirely” (Rosenberger, 2020, p. 135). Examples include installing excessive armrests on benches, spikes on windowsills, and large planters in spaces that could otherwise help people congregate or rest. These alterations to public spaces, created in the name of public safety, actually expose community members to more danger, namely by depriving them of access to places to rest, eat, congregate, and seek resources. Outwardly, hostile architecture may signal safety to members of dominant groups, while in the process rendering marginalized groups even more vulnerable by exiling them further to the margins of social life, given how hostile design features “push the unhoused and others out of public spaces, out of safety, out of the community, and out of view” (Rosenberger, 2020, p. 148). Moreover, investments in hostile architecture are made at the expense of investments in programs and services designed to promote safety. Ultimately, hegemonic public safety discourses alter spaces by dictating which bodies are granted and ensured access into public life. Public and private gatekeepers use various means to communicate these terms and conditions for participation in public life, including hostile architecture and policies whose violations are punishable by fines and imprisonment.

### 4.9 Summary

Taken together, hegemonic public safety discourses do the work of governmental bodies as well as the bidding of dominant groups. Public safety gets weaponized in service of anti-Blackness and therefore distorted into a pretense for vigilante justice and other harmful forms of surveillance. Hegemonic public safety discourses also help to legitimize the manufactured need for policing and incarceration, at the expense of investing in programs that would help reduce and eliminate harm and danger in our families, neighborhoods, and organizations. Given how entrenched these discourses are within our culture, is it possible to reclaim public safety? These consequences of neoliberal, carceral approaches to public safety echo the words of Barbara Smith: “You cannot be safe under systems of mass oppression” (quoted in Page and Woodland, 2023, p. 29). Given these constraints, how might we resist mass oppression in order to promote greater safety for *everyone* in our communities, especially those who are most harmed by punitive policies promoted in the name of public safety?

## 5 A different approach: life-affirming public safety frameworks

As described in the preceding sections, hegemonic public safety frameworks are rooted in carceral logics. To counter these carceral logics, we draw on Collins (2007) practice of “shifting the center” as an entry point for divesting public safety from carceral frameworks. In the following sections, we describe how “shifting the center” can open new pathways toward justice. Next, we illustrate how healing justice as a lineage and lens can help to identify and counter the carceral logics embedded within hegemonic public safety discourse. We conclude with a roadmap for an alternative approach to carceral logics: life-affirming safety frameworks.

### 5.1 Shifting the center in public safety discourse

Feminist theorist Patricia Hill Collins (2007) invites us to engage in a process of “shifting the center” (p. 311), whereby we transform our understanding of taken-for-granted phenomena by diverting the spotlight away from the ruling class and instead toward groups that have been silenced and criminalized. To do so, Collins urges us to “distinguish between what has been said about subordinated groups in the dominant discourse, and what such groups might say about themselves if given the opportunity … [to] create new themes and angles of vision” (p. 314). In this piece, we have attempted to do just that: identify what has been said about subordinated groups (namely, those which are subject to surveillance and policing) in the dominant discourse around public safety. Inspired by Collins’ call to “shift the center,” we invite readers to (re)imagine public safety from the perspective of community members who are most harmed by hegemonic public safety discourses. How does our understanding of public safety change when we “shift the center” away from the ruling class and instead toward people whose needs have been deprioritized, ignored, and/or endangered within dominant public safety frameworks? How will this shift invite new themes and angles of vision? And by shifting the center, how can we cultivate liberation for the communities in most urgent and dire need of justice?

We believe that shifting the center in public safety discourses opens new pathways toward justice without exacerbating existing inequities within our society. Justice is often conflated with participation in the criminal legal and family regulation systems, which are inherently carceral and inequitable. Shifting the center allows us to think against and beyond carcerality—and to instead approach justice as the process of repairing and transforming interpersonal and institutional harm. Striving for justice requires us to make amends for the ways that systems of oppression have enabled the concentration of power, resources, and security among the ruling class (Jeffries-Logan et al., 2016). Justice work inspires interventions that reduce and prevent harm by addressing the root causes, as well as responses that help people restore their sense of safety and wholeness when they do experience harm or violence (Haines, 2019).

The practice of shifting the center can be a powerful guide in justice work by changing our understanding of who is deserving of support, whose safety needs are most critical and urgent, where the onus of responsibility falls, and how resources ought to be redistributed. Shifting the center will also help resource and empower community groups which are already working to promote public safety without leaving anyone behind. Therefore, shifting the center is a valuable tool for (1) analyzing the harms embedded within hegemonic public safety discourses and (2) interrupting and transforming these dominant discourses. This shift is desperately needed to cultivate a sense of safety—as well as actual public safety—for *all* members of society, especially those who are deemed dangerous or unworthy of protection under hegemonic public safety discourses.

### 5.2 Reconceptualizing safety through healing justice

To guide us in shifting the center in public safety discourse, we draw upon alternative models that contest, reject, and transform the carceral logics embedded within dominant paradigms. Of particular relevance to psychologists and practitioners is the notion of *healing justice*, a framework that “seeks to intervene on generational trauma to build collective power towards resistance” (Claudia Lopez, as quoted in Page and Woodland, 2023, p. 138). Healing justice is a direct response to the physical, psychic, symbolic, material, and intergenerational harms from systemic injustices including criminalization, surveillance, forced displacement, policing, and other forms of state violence. Instead of replicating carceral responses, healing justice uses alternative strategies that are rooted in, and in service of, “transformative justice, disability justice, reproductive justice, environmental justice, and harm reduction” (Page and Woodland, 2023, p. 137).

Similar frameworks include the care-based approach to public safety (Norris, 2020), nurturance culture (Samaran, 2019), an ethics of care (Robinson, 2011), and cultural frameworks (Singer et al., 2021). What these frameworks share is a firm commitment to dismantling the conditions that create a sense of safety for dominant groups at the expense of justice and well-being for marginalized communities, including capitalism, carcerality, racism, and ableism (Haines, 2019; Norris, 2020; Hamaji et al., 2021; Page and Woodland, 2023). Healing Justice frameworks invite us to reimagine safety as a community-based (vs. state-controlled) endeavor that is rooted in collective care (vs. individual self-preservation), affirms and preserves human life (vs. property), values autonomy (vs. control), promotes harm reduction (vs. criminalization), and works toward liberation (vs. retribution). A healing justice approach understands safety as a “community-determined, survivor-centered process that does not rely on policing and punishment [and] refers to how we navigate our different needs and desires based on lived experience and individual and collective trauma while preventing, minimizing, and transforming harm, violence, and abuse” (Page and Woodland, 2023, pp. 277–278). In this way, healing justice has much to offer for helping us identify hegemonic public safety discourses, redress the consequences of carceral public safety frameworks, and shift the center in public safety interventions.

Given its roots in anticapitalism and prison abolition, healing justice is by nature a collective project that must be enacted beyond the confines of the state apparatus. Healing justice is also a simultaneous retrospective and projective project that guides us in looking forward to (re)create a new world while also looking backward to reclaim ancestral ways of beings, given how policing and prisons are relatively recent artifacts of U.S. settler colonialism and chattel slavery (Morris, 2023). Put simply, people have already created safe communities without policing, punishing, and imprisoning each other, which means we have the collective power to do so again (Morris, 2023).

Just as an abolitionist lens calls upon us to look both backwards and forwards in time, so too does it invite us to participate in simultaneous creation and destruction. Abolition involves the complementary projects of decarceration (dismantling carceral structures) coupled with the creation of alternatives which sustain life and create the conditions necessary for all communities to survive and thrive. Life-affirming interventions are those which seek to preserve and improve the health, safety, and overall wellbeing of *all* groups, particularly in the context of social institutions such as education, healthcare, and housing (Ruth Wilson Gilmore, as quoted in Davis et al., 2022, p. 51). If carceral frameworks are those which elevate the likelihood of and intensify punishment for interpersonal harm, then life-affirming frameworks are those which seek to lessen and prevent the likelihood of harm by improving the material conditions for all people. How, then, would public safety discourses and policies change if we made a collective commitment toward building life-affirming institutions?

### 5.3 Building life-affirming safety infrastructure

Various social movements and projects have proven how shifting the center in mainstream public safety discourse is a matter of life-and-death for communities that are endangered via their criminalization and vulnerability to state violence. Throughout time, vulnerable communities have taken their safety and healing into their own hands, given their inability to rely on the state and other established yet inadequate sources of support.

- To reduce racial profiling in traffic stops, improve vehicular safety, and lessen the likelihood of traumatic and potentially lethal interactions with law enforcement, groups such as NorCal Resist have offered free headlight/taillight repair clinics.
- To mitigate street harassment and gaybashing on public transportation and taxis, groups like Homobiles organized pay-what-you-can rideshares to help reduce isolation and facilitate safety through numbers.
- To prevent and help revive people from drug overdoses, autonomous and formalized groups alike have operated safer drug consumption and delivery sites, harm reduction supply distributions, needle exchanges, and Narcan administration trainings.
- To lower the risk of assault and financial exploitation, sex workers circulate “bad date lists” to warn one another about abusive clients.
- To minimize encounters with law enforcement, people have compiled guides for supporting people through various crises without calling the police, including suicidality, medical emergencies, vehicle breakdowns, and domestic violence (e.g., Boyd et al., n.d.).
- To help people develop the skills needed for community self-defense and physical safety, organizations like Harriet’s’ Wildest Dreams in the DMV area and Ujimaa Medics in Chicago provide their neighbors with critical survival skills including stop-the-bleed trainings and mental health first aid.
- To counter the symbolic and material violence associated with enduring poverty and homelessness, autonomous groups redistribute food, offer street medicine, and beautify hostile architecture.
- To (re)build robust networks of care in the aftermath of abuse (as well as in attempts to minimize abuse altogether), informal networks of care engage in accountability practices such as podmapping (Mingus, 2016) and Madmapping (The Icarus Project, 2015), which help people identify who they might connect with in the event that they experience and/or cause interpersonal harm, or find themselves in some sort of crisis.

These life-affirming experiments—varying in scope, scale, tactics, and longevity—serve as proof of concept that noncarceral approaches to public safety not only are possible, but also exist already and will continue to remain and regenerate over time. Moreover, these noncarceral approaches help affirm and sustain life by countering the punitive logics of carceral public safety frameworks.

In summary, life-affirming frameworks can help to identify, challenge, and transform hegemonic public safety discourses by dispelling racialized crime mythology (including permanent bad guy syndrome), reconciling the victimization-fear paradox, and rejecting the politics of ideal victimhood. Life-affirming frameworks can also inspire social transformations that are not possible under carceral public safety frameworks, such as elevating the safety concerns of groups that are intentionally rendered marginalized and under-resourced, creating pathways for stigmatized communities to seek care and support in the aftermath of targeted violence, and identifying and dismantling state structures which create and exacerbate public health concerns. In sum, these life-affirming frameworks can guide us toward decarcerating dominant paradigms, practices, and policies in the realm of public safety.

## 6 Conclusion

In the outset of this paper, we referenced Mayor Thao’s State of the City speech, wherein she discussed public safety in ways that many mayors often do—by coupling public safety with policing. As people committed to creating a world where everyone can feel and be safe, we have concerns with strategies that respond to safety concerns with tactics that further endanger neighbors who are already vulnerable to interpersonal and institutional violence. We are also concerned with tactics that invest public resources into projects that threaten, rather than affirm and sustain, all human life.

In the aforementioned State of the City speech, Mayor Thao proudly boasted that, “in this year’s budget, we made the city of Oakland’s largest ever investment in housing” (City of Oakland, 2023). This is important, because Oakland is in a county with deep poverty. It experiences one of the highest rates of wealth and income inequality in the nation (Bay Area Council Economic Institute, 2021) and classifies close to half of its residents as housing burdened (National Equity Atlas, 2020). Curiously, the Mayor explicitly framed housing and homelessness as separate issues from public safety (which was almost exclusively equated with policing). Our overview of carceral public safety frameworks through the lens of abolition helps reveal at least four problems that arise from this separation between public safety and housing (and poverty more broadly). First, the framing detracts attention from the ways that housing insecurity, including homelessness, is in and of itself an urgent public health and safety concern (Barocas et al., 2023; Goldshear et al., 2023). Not surprisingly, those facing housing insecurity are less frequently ideal victims, making it easier to separate the issue from broader discussions of public safety within the carceral public safety rubric. Second, by appealing to the ideal victim archetype, The Mayor’s framing starkly removes the more than 5,000 Oakland residents enduring homelessness from consideration when city leaders determine which subgroups are deserving of greater safety (Applied Survey Research, 2022).

Third, the framing detracts attention from the ways that the city, county, state, and country are complicit in creating and exacerbating the conditions that lead to housing insecurity and, by extension, a myriad of other downstream public health and safety concerns. Because the city, and by extension the state, is generally not named in discourse on public safety, this separation also makes it easier to exclude issues of housing insecurity from discussions of public safety under carceral public safety frameworks. Finally, this framing helps justify the expansion of carceral approaches at the expense of life-affirming responses. Specifically, the city’s leadership has chosen to address public safety concerns by increasing police presence and policing technologies (e.g., automated license plate readers), while minimally investing in life-affirming responses. These policy decisions have positioned Oakland as one of the most highly funded police departments in the country. More than 20% of the entire city budget has been devoted to policing, which City Council topped-off using more than 40 % of the general fund, resulting in a nearly $800 million investment in policing (BondGraham, 2021)—a staggering amount of money especially given the significant concerns associated with relegating public safety to the purview of police.

In contrast, a care-based, life-affirming approach to public safety immediately links poverty and its correlates to public safety, and therefore advocates for allotting resources toward the elimination of poverty as a front-line approach to creating public safety. This (re)distribution of resources includes investments in “healthcare, mental health services and treatment, educational opportunities, affordable housing, transit access, and investments in youth” (Hamaji et al., 2021, p. 81). A care-based, life-affirming approach also works directly with people experiencing housing insecurity to ensure that their safety needs are understood and taken seriously. Such an approach would transform the collective imagination around the criminalization of poverty, such that vulnerable groups are not regarded as threats to privileged people’s public safety but rather agents of public safety who themselves are unconditionally deserving of safety and care.

Ultimately, we echo calls to decouple public safety and policing practices. Contesting carceral approaches to public safety will ensure that we as a society are investing resources into communities to help them thrive, because thriving communities are safe communities. Reorienting our vision of public safety away from law enforcement, and the state apparatus more generally, will help create the conditions necessary for all communities to flourish, especially those most harmed by the carceral public safety framework.

## Author contributions

VR: Writing – review & editing, Writing – original draft, Project administration, Conceptualization. OS: Writing – review & editing, Writing – original draft, Project administration. WS: Writing – review & editing. SF: Writing – review & editing.
